# Efficient Photo-Driven Electron Transfer from Amino Group-Decorated Adamantane to Water

**DOI:** 10.3390/molecules30163396

**Published:** 2025-08-16

**Authors:** Xiangfei Wang, Jonathan Remmert, Beate Paulus, Annika Bande

**Affiliations:** 1 Institute of Chemistry and Biochemistry, Freie Universität Berlin, Arnimallee 22, 14195 Berlin, Germany; b.paulus@fu-berlin.de; 2 Helmholtz-Zentrum Berlin für Materialien und Energie, Hahn-Meitner-Platz 1, 14109 Berlin, Germany; annika.bande@aci.uni-hannover.de; 3 Department of Physics, Freie Universität Berlin, Arnimallee 14, 14195 Berlin, Germany; jonathan@remmert.berlin; 4 Institute of Inorganic Chemistry, Leibniz University Hannover, Callinstr. 9, 30167 Hannover, Germany

**Keywords:** adamantane, amino group, electron transfer, excited states, linear response time-dependent density functional theory (LR-TDDFT)

## Abstract

Nanodiamonds in water can generate solvated electrons under ultraviolet (UV) excitation, but UV light constitutes only a small portion of solar energy. To harvest solar energy in the visible range, we investigate band gap reduction via surface amino functionalization and examine its impact on photo-excited charge transfer to water. Adamantane, the smallest nanodiamond, is used as a model due to its electron emission properties. Liquid water is first represented using water dimers and then complete solvation shell structures surrounding the adamantane. By systematically analyzing different functionalized adamantane structures, we find that nitrogen serves as the primary electron donor to nearby water molecules. Furthermore, the negative electron affinity of adamantane, which determines its emission capability, is preserved with half of the amino group functionalization on the surface. Our findings motivate further experimental verification using nanodiamonds with amino-functionalized surfaces.

## 1. Introduction

With the development of modern society, there is a growing demand for energy due to human activities. Currently, the primary source of energy relies on fossil fuels. Their combustion releases a large amount of ${\mathrm{CO}}_{2}$, which is one of the leading causes of the greenhouse effect and, consequently, climate change [1]. To use energy in a sustainable way, research on photochemistry has been focusing on designing materials that can harvest energy from sunlight. One of the most promising candidates is the nanodiamond (ND), which has attracted considerable attention [2] due to its unique electronic properties [2], eco-friendly composition [3,4], and increasing availability through modern synthesis techniques [5,6,7,8]. Therefore, there has been widespread research into the application of NDs for generating green $H_{2}$ gas [9] from solar energy and for the reduction of ${\mathrm{CO}}_{2}$ [10] and $N_{2}$ [11].

To understand NDs as effective catalysts for photochemistry, it is important to investigate their surface structure. NDs have an exceptionally high surface-to-volume ratio [12], providing abundant sites for chemical reactions. In addition, the surface of NDs allows for various functionalizations. The most common type of surface termination involves hydrogen atoms, which enable high chemical stability and resistance to degradation [13,14]. Furthermore, the hydrogen atoms on the surface carry a positive charge, while the nearby carbon atoms are negatively charged. The resulting charge separation creates dipole moments, leading to negative electron affinity (NEA) [15,16]. NEA is a property shared by all forms of NDs down to the smallest adamantane ($C_{10}H_{16}$). Specifically, when NDs absorb photon energy, electrons are excited to the conduction band and subsequently migrate to the surface. Due to NEA, the emission process can be highly efficient [15,17,18]. Therefore, the applications of NDs range from fluorescent imaging [19] to field emission displays [20]. In particular, recent experimental and theoretical studies [21,22] show that illuminated NDs in a water environment are an effective source of solvated electrons, which can activate a variety of high-energy reduction reactions, such as the reduction of $N_{2}$ to ${\mathrm{NH}}_{3}$[11] and ${\mathrm{CO}}_{2}$ to CO [23]. Understanding the electron emission process into water is critical for the design of illuminated NDs as efficient catalysts for harvesting solar energy.

To investigate the mechanism of photo-excited electron transfer from NDs to liquid water, Wang et al. [24] used adamantane to represent NDs and small water clusters to model the electronic properties of liquid water. Using linear-response time-dependent density functional theory (LR-TDDFT), their calculations show that the photo-excited electron transfers from adamantane to various electronically bound states of the surrounding water clusters. Although the surface plays a determining role in the electron emission properties of NDs, the effects of functional groups remain less explored. Buchner et al. [21] showed that carboxyl and carbonyl terminations can suppress the emission of solvated electrons from NDs into liquid water. Kirschbaum et al. [25] suggested that amino functional groups can reduce the band gap of hydrogen-terminated NDs (approximately 5.4 eV) [16] to the visible range of the solar spectrum (1.6–3.3 eV) [26]. However, how amino functionalization determines the electron emission from NDs into liquid water remains unclear.

Following a similar approach, this study uses water clusters as models for the electronically bound states in liquid water. In particular, the simplest water dimer with $C_{s}$ symmetry [27] is sufficient to host an electronically bound state. Furthermore, adamantane is used as a model for NDs due to its negative electron affinity (NEA) and similar electron emission properties. To understand the role of nitrogen atoms, various amino group-decorated adamantane structures are studied with respect to their frontier orbital energies, which reflect their ionization and NEA properties. Additionally, different relative positions of the water dimer are systematically explored around the amino-decorated adamantane. To consider a more complex solvation environment involving hydrogen bond networks, we also use molecular dynamics simulations to sample the solvation shell structures. By systematically exploring water models of increasing accuracy, we reveal the mechanism of photo-induced charge transfer into liquid water. Electronic structure calculations further clarify how amino-functionalized surfaces determine the photo-excited charge transfer process.

## 2. Results

### 2.1. Electronic Properties of Amino-Functionalized Adamantane

**Figure 1:**
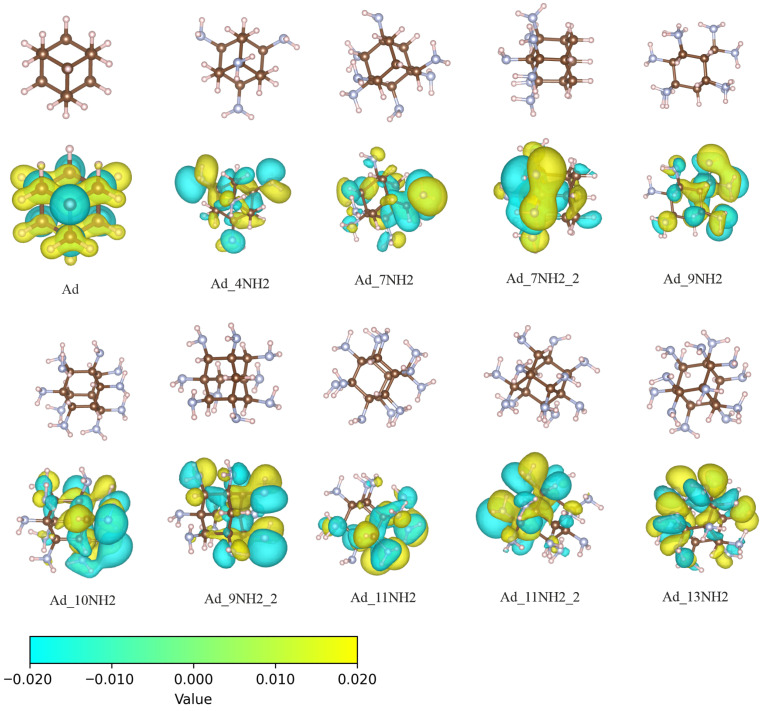
The structures as well as the HOMOs of adamantane with varying amino surface functionalization are shown. The upper row displays only the structures, while the lower row presents the HOMO of the corresponding structure. The naming convention for these structures includes “Ad” for adamantane followed by the number of amino groups. The final segment in each name distinguishes different isomers. The isovalue for all orbital plots was set to $2\times 10^{-2}\;a_{0}^{-3/2}$. The bottom of the figure shows the color bar corresponding to the isovalue contour plot. The yellow and cyan colors represent the positive and negative values of the wave function, respectively.

There are multiple possibilities for adding amino groups to adamantane; we selected the 10 systems presented in Figure 1 to model different chemical situations. To introduce the amino functional group, instead of increasing the number one by one, we added four amino groups at once and arranged them at equivalent positions on the surface. The functionalization sites were in a tetrahedral configuration compatible with the $T_{d}$ symmetry group of adamantane. Since the four positions were equivalent, the symmetric structure served as a good representation of an evenly functionalized adamantane surface. Additional structures were systematically constructed based on this symmetrically modified structure. The total number of amino groups increased from 4 to 13. For the same number of amino groups, we selected representative isomers with distinct structures for investigation. A complete enumeration of all possible isomers was beyond the scope of this study.

The electrons in the highest occupied molecular orbital (HOMO) are the most active ones in chemical processes [28]. In particular, the ionization potential theorem of Kohn–Sham density functional theory (KS-DFT) establishes that the negative value of the HOMO energy is an approximation of the ionization energy [29,30,31]. Therefore, during the photoexcitation process, the electron is first removed from the HOMO. Thus, the position and shape of the HOMO provide a qualitative analysis of different molecular segments as electron donors. Figure 1 shows the structure of adamantane together with the systematically explored amino-functionalized structures. Beneath each structure, the HOMOs are also depicted accordingly. For adamantane, the HOMO localized on the carbon atoms. For amino group-functionalized structures, the HOMO localized primarily on the nitrogen atoms. One particular structure is Ad_7NH2_2, which has all the nitrogen atoms on the same side. Accordingly, the HOMO was also primarily located on the same side of the Ad_7NH2_2 molecule.

**Figure 2:**
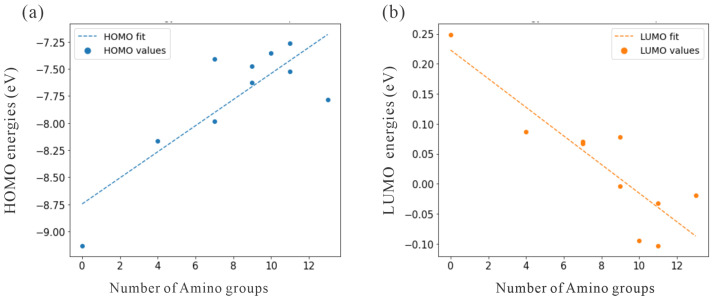
Dependence of HOMO and LUMO energies on the number of surface amino groups in functionalized adamantane. (**a**) The HOMO energy (in eV) as a function of the number of amino groups. The dashed line shows a linear fit. (**b**) The LUMO energy versus an increasing number of amino groups. Legend and units are the same as in (**a**).

To illustrate the role of nitrogen atoms as electron donors, we analyzed the HOMO energies. Table 1 shows that from adamantane to Ad_4NH_2, the amino group increased the HOMO energy from −9.131eV to −8.162eV. Furthermore, the HOMO energies continued to increase with a higher number of amino groups, as shown by other structures. This trend, shown in Figure 2a, confirms that amino groups effectively reduced the ionization energy, thereby facilitating the photoexcitation process. To avoid unintentional excitation of the water, we compared the HOMO energies of the water. For the water monomer and small water clusters, the CAM-B3LYP/aug-cc-pVDZ level of theory predicted the HOMO energy to be approximately −10.5eV [24]. Furthermore, the experimentally measured ionization energy for liquid water was about 9.9eV [32,33]. Because the HOMO energies of water in different states of matter were lower than those of amino-functionalized adamantanes, the water was not excited when amino-functionalized adamantane was available for excitation.

For an estimate of the electron affinities of the amino-functionalized adamantane structures under investigation, we used the LUMO energies. Table 1 shows that the LUMO energies decreased with an increasing number of amino groups. In particular, the LUMO energy of adamantane was 0.249eV, and that of Ad_4NH2 was 0.087eV. The energies of both structures were above zero and therefore corresponded to an NEA. However, with a higher number of amino functional groups, some structures exhibited negative LUMO energies, including Ad_9NH2, Ad_10NH2, Ad_11NH2, Ad_11NH2_2, and Ad_13NH2. For a direct visualization of this trend, Figure 2b shows that the LUMO energies decreased systematically with an increasing number of amino groups. Consequently, the original NEA property of adamantane was reduced with a higher degree of amino functionalization. Nevertheless, most of the amino-functionalized structures had positive LUMO energies. Therefore, the photo-excited electron will be attracted to the water molecules rather than the differently decorated adamantane structures, which have a lower electron affinity.

To explain the loss of NEA properties upon amino functionalization, we performed Hirshfeld population analysis on all structures to determine the atomic charges. For each atom type, we averaged the charges over the same atoms. As shown in Table 1, the pure adamantane had carbon atoms with a charge of −0.122 and surface hydrogens with a charge of +0.082. The surface dipoles pointed uniformly from hydrogen to carbon, resulting in NEA. Introducing amino groups (e.g., Ad_4NH_2_) reduced the negative charge on carbon and instead places a larger negative charge on nitrogen (−0.380). This is because nitrogen has a higher electronegativity than carbon. In particular, hydrogen had the lowest electronegativity and remained positively charged in all structures. With more than four amino groups, some carbon atoms became positively charged, disrupting the original surface dipole. Therefore, amino-functionalized structures feature dipoles that point from hydrogen to nitrogen in various directions and result in the loss of the NEA property.

### 2.2. Charge Transfer to Water Dimers

**Figure 3:**
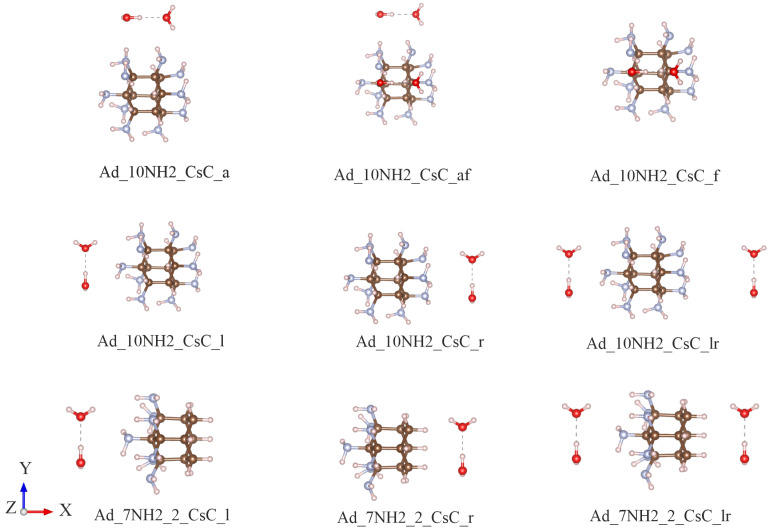
Schematics showing the structures used to calculate electron transfer from amino-functionalized adamantane to water molecules. $C_{S}C$ water dimers are depicted in various positions and orientations relative to adamantane: above (a), in front (f), to the left (l), and to the right (r). Additionally, two types of amino-functionalized adamantane were considered, namely Ad_10NH2, with nitrogen atoms uniformly distributed, and Ad_7NH2_2, with nitrogen atoms concentrated on one side.

**Figure 4:**
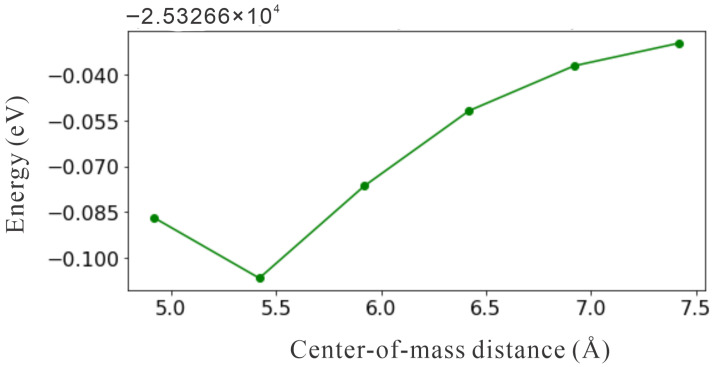
The total ground state energy as a function of the center-of-mass (COM) distance between the water dimer and the Ad_7NH2_2 cluster in the Ad_7NH2_2_CsC_l structure is shown. The energy is given in eV units, and the COM distance is in Å units.

To study the photo-excited charge transfer to the liquid water, the water dimer was used as an effective representation of the electronic bound state. The most stable water dimer had the $C_{S}$ symmetry with the hydrogen atoms in a cis configuration ($C_{S}C$) [27]. For the amino-functionalized adamantane, two specific structures were considered: one with amino groups on a single side (Ad_7NH2_2) and another with amino groups uniformly distributed across the entire surface (Ad_10NH2). Regarding the relative geometry effects, this study investigates various placements and orientations of the $C_{S}C$ water dimer. Even though the number of possible relative geometries can be numerous, only select systematic structures were considered. Figure 3 shows the representative structures in which the $C_{S}C$ water dimers were arranged around adamantane. For each orientation, the optimal center-of-mass (COM) distance between the water dimers and adamantane was set to minimize the total system energy. As an example, Figure 4 shows the energy as a function of the center-of-mass (COM) distance for the Ad_7NH2_2_CsC_l structure. The minimum energy occurred at a COM distance of 5.420 Å, which defines the optimal separation of the water dimer relative to the functionalized adamantane. The structure details are in Appendix A.

LR-TDDFT calculations were performed on all joint structures to obtain various excited states. Although all excited states up to 20 were computed, this study focused on those in which more than 90% of the hole density was localized on amino-functionalized adamantane. Among these selected states, only the lowest-energy excited state was analyzed in detail to investigate the spatial distribution of the photo-excited electron. Nevertheless, due to the presence of an electronic bound state associated with the water dimer, all of the preselected states exhibited partial charge transfer to the water dimer. Therefore, in LR-TDDFT calculations, the lowest excited states with a hole only on the adamantane were referred to as the lowest-charge transfer states.

To demonstrate that photoexcitation and electron transfer to water molecules are feasible, Table 2 provides a quantitative analysis of the lowest-charge transfer states. For each of the structures, there was consistent partial charge transfer ($q_{w}>0$) to the $C_{S}C$ water dimers. Therefore, the photo-excited electron was, in any case, transferred to the water dimer, regardless of position and different surface functionalizations. However, all the states exhibited incomplete charge transfer ($q_{w}<1.0$) due to the low electron affinity of the $C_{S}C$ water dimer. For complete charge transfer with $q_{w}$ close to 1.0, the water cluster must have a high electron affinity and form deep electronic bound states.

After confirming the charge transfer to the water dimer, the role of nitrogen atoms as electron donors needed to be explored. Table 2 shows that the electron NTO density minus the hole NTO density for all nitrogen atoms ($q_{ad}^{N}$) was negative. Therefore, the nitrogen atoms primarily contributed to the hole of the exciton. In addition, the absolute value of $q_{ad}^{N}$ was larger than that of $q_{w}$. The large magnitude of $q_{ad}^{N}$ indicates that charge transfer primarily occurred from the nitrogen atoms to the water molecules. Consequently, a small portion of the charge was transferred from the nitrogen atoms to the carbon atoms. The contribution of nitrogen atoms in donating electrons to water can be verified by the shape of the HOMO, which was primarily localized around the nitrogen atoms. In addition, the charge transfer states were all optically accessible with significant transition dipole moments.

The charge transfer excited states had an excitation energy of roughly 5 eV, which is about 2 eV lower than the absorption onset of pure adamantane at approximately 7 eV [24]. Similarly, large nanodiamonds exhibit excitation energies near 5 eV [34]. Thus, amino functionalization of nanodiamond surfaces could potentially lower the charge transfer excitation energy to about 3 eV, which lies in the visible range of the spectrum.

Finally, the last column of Table 2 shows that most charge transfer states corresponded to the first excited state. The association of charge transfer states with the lowest excited state is important, as higher excited states will rapidly relax to the lowest excited state. In the first example, the highly functionalized Ad_10NH2_CsC_l exhibited charge transfer at the second excited state due to the reduced electron emission properties caused by the excess amino groups. For another example, Ad_7NH2_2_CsC_r showed its charge transfer at its third excited state. Here, the amino groups were primarily on the left side of the structure, while the water dimer was on the right. Therefore, the geometric arrangement is unfavorable for charge transfer. The influence of geometry will be explored further in the following section.

### 2.3. Charge Transfer to Water Dimers in Proximity

**Figure 5:**
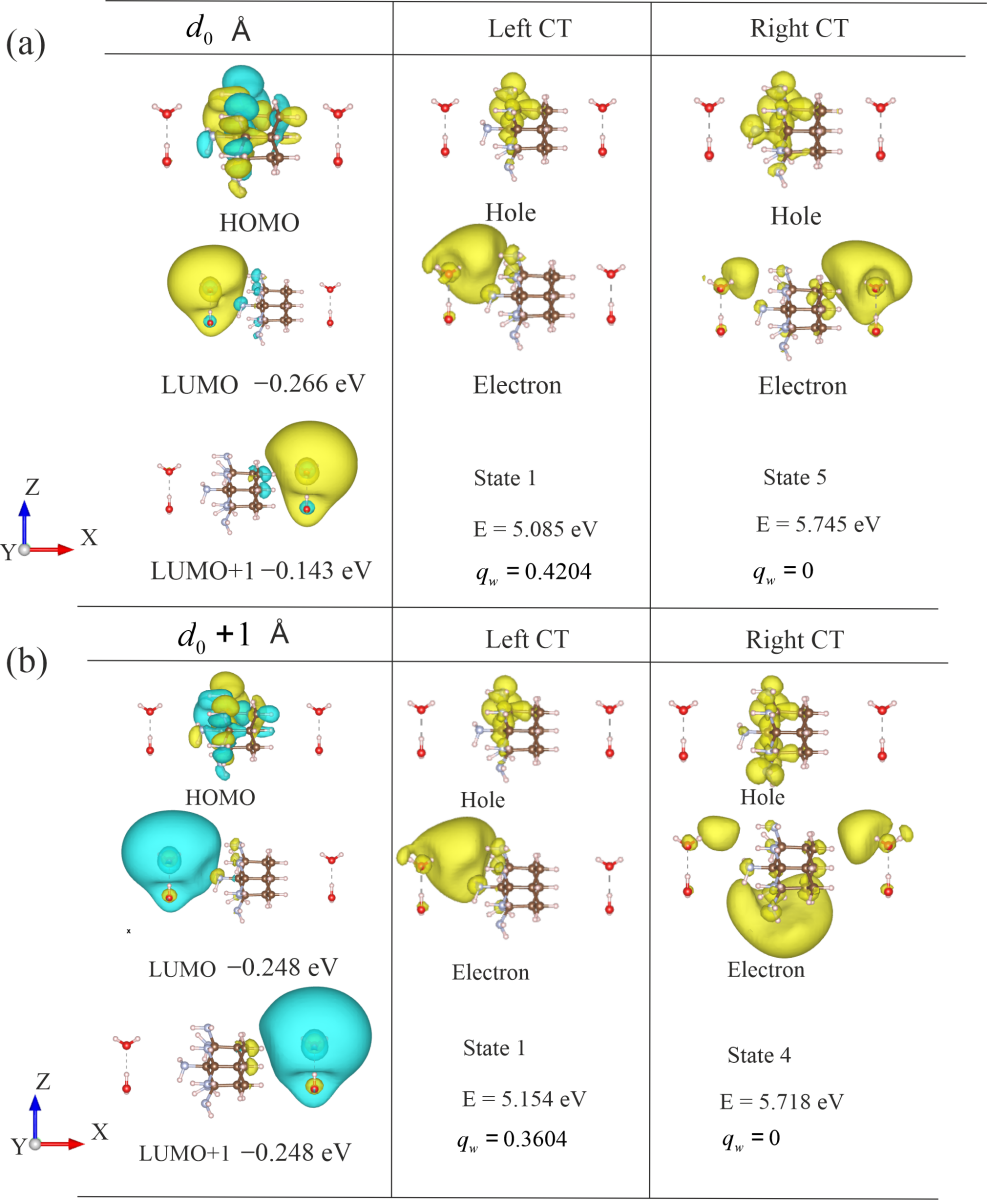
Photo-excited charge transfer from amino-functionalized adamantane to water dimers at various distances. The structure corresponds to the Ad_7NH2_2_CsC_lr configuration, as shown in Figure 3 in this study. (**a**) The left and right water dimers at their respective equilibrium distance $d_{0}$. (**b**) The structures after shifting both the left and right water dimers 1 Å further away from their respective equilibrium distances. The left column displays the molecular orbitals, including the HOMO, LUMO, and LUMO+1. The middle and right columns show the NTO densities for the left and right charge transfer states. Qualitative properties, including the order of the excited state, lowest charge transfer excitation energies ($E_{ct}$), and charge transferred to the water molecules ($q_{w}$), are also provided alongside the corresponding NTO plots. The isovalue for all orbital plots was set to $2\times 10^{-2}\;a_{0}^{-3/2}$. The isovalue for the NTO plots was set to $4\times 10^{-4}\;a_{0}^{-3}$.

In the previous section, we found that nitrogen atoms can donate electrons during photoexcitation. Therefore, it is important to understand whether the water molecules adjacent to the amino functional groups are more likely to attract the electron than the water dimer that is farther away. To investigate the effect of different relative positions, the one-sided amino-functionalized adamantane molecule Ad_7NH2_2 is used as an example. To compare the influence of water molecules at different distances relative to the nitrogen atoms, two identical water $C_{S}C$ clusters were placed symmetrically on the left and right sides. First, the water clusters were arranged at their respective equilibrium distances ($d_{0}$). Then, the two water clusters were systematically shifted away from their equilibrium positions. Figure 5 shows the structures, molecular orbitals, and lowest charge transfer excited-state properties for the left and right water dimers at different distances.

At the equilibrium distance, the water dimer interacted strongly with the surface of adamantane. This influence is reflected in the molecular orbitals and their energies. Specifically, the left panel of Figure 5a shows the HOMO, LUMO, and LUMO+1 of the system with two water dimers at a distance $d_{0}$. The HOMO was localized on the nitrogen atoms of the decorated adamantane, while the LUMO and LUMO+1 were primarily located on the left and right water dimers, respectively. Although both water dimers had identical structures, the left water dimer interacted more strongly with the nitrogen atoms, leading to a lower orbital energy. In contrast, the right water dimer was farther from the nitrogen atoms and consequently had a higher orbital energy. The charge transfer exciton featured orbital transitions primarily involving the HOMO-to-LUMO transition. For example, the charge transfer state to the left water dimer involved an orbital transition from the HOMO to the LUMO. The charge transfer state to the right water dimer involved a transition from the HOMO to the LUMO+1. In addition, the magnitude of charge transfer to the left water dimer was 0.420, and the charge transfer to the right water dimer was 0.413. The excitation energy of the left charge transfer state was also lower than that of the excited state corresponding to the right water dimer. Therefore, the photo-excited electron transfer was more favorable to the left water dimer, which was closer to the electron-donating nitrogen atoms.

The distances between the water dimers and the nitrogen atoms play a crucial role in the charge transfer process. Therefore, it is important to verify the charge transfer behavior at different distances. For example, when the water dimers were shifted 1 Å further away from their original positions, the influence of the nitrogen atoms decreased. Figure 5 shows the lowest charge transfer state with the distance increased to $d_{0}+1$ Å. As shown in Figure 5b, when the water molecules moved away from the amino-functionalized adamantane, the HOMO and LUMO of the two charge transfer states had similar shapes to the molecular orbitals in Figure 5a. Nevertheless, due to the larger distance, the transferred charge ($q_{w}$) from the amino-functionalized adamantane to the water dimer decreased, and the energy required for excitation increased. Consequently, charge transfer became more difficult at longer distances. The charge transfer excited state to the right water dimer was particularly sensitive to longer distances. The magnitude of the charge transferred to the right water dimer was only a fractional value of 0.160. Therefore, the photo-excited electron had a distribution predominantly around the amino-functionalized adamantane, which means that charge transfer to the right water dimer could be challenging.

For structures with greater distances, we summarize the charge transfer state properties in Table 3. The table includes the magnitude of the transferred charge ($q_{w}$) to the water dimers, the excited state energies (*E*), as well as the order of the excited states. The distance systematically increased from $d_{0}$ to $d_{0}$ + 4.0 Å. The charge transfer state exhibited a consistent trend favoring the left water dimer. Specifically, the excited state with charge transfer to the left water dimer had a lower excitation energy than the charge transfer state to the right water dimer. Furthermore, the magnitude of transferred charge to the left water dimer was also systematically larger than that to the right water dimer. Therefore, the photo-excited charge transfer was more favorable to the water cluster that was in close proximity to the nitrogen atoms. Furthermore, the charge-transfer state involving the left water dimer consistently corresponded to the first excited state, resulting in a monotonically decreasing charge transfer $q_{w}$ with increasing distance. In contrast, the charge transfer states associated with the right water dimer had higher excitation energies, typically the fourth or fifth state. Therefore, the photo-excited charge transfer favored the left water dimer due to the lowest excitation energy and close proximity.

### 2.4. Charge Transfer to Water Molecules Within the Solvation Shell

As shown previously with simple water dimers, charge transfer favored water molecules near the amino groups. However, the solvation environment around adamantane is far more complex than that in a dimer model. First, liquid water forms an extended hydrogen bond network with bond breaking and reforming, which can influence the charge transfer [35]. Although a dimer already contains a broken hydrogen bond, it does not fully capture the influence of the extended network. Second, water molecules can form hydrogen bonds directly with the amino groups, generating unique hydration structures [36]. Finally, DFT ground-state optimizations yield 0 K geometries and neglect thermal fluctuations, which may also affect charge transfer.

To sample more realistic hydration structures, we performed MD simulations on the one-sided functionalized Ad_7NH2_2 system. Three snapshots were selected at random for excited-state calculations. In all cases, the lowest excited state showed charge transfer to the solvation shell close to the amino groups. The excitation energies were 5.258, 5.552, and 5.792 eV, and the charge transferred to water $q_{w}$ was 0.943, 0.959, and 0.933, respectively.

**Figure 6:**
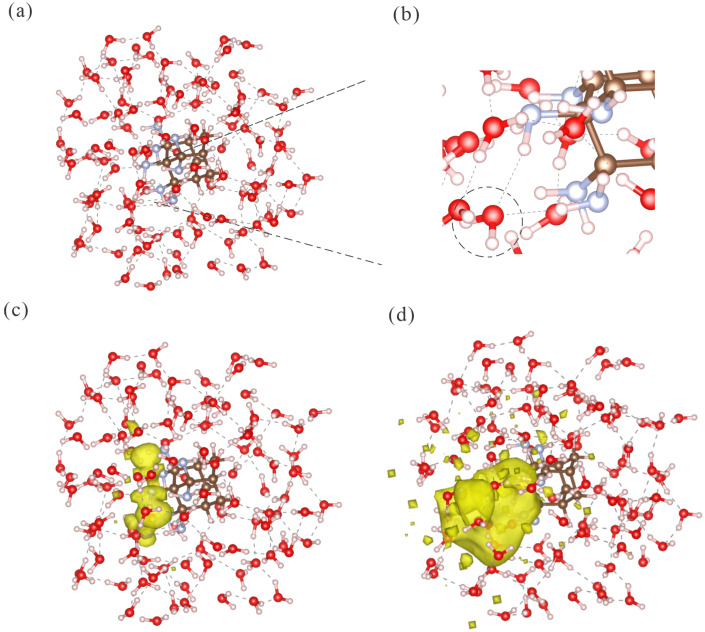
Solvation shell structures and charge transfer excited-state properties of one exemplary MD snapshot. (**a**) The water solvation structure around the amino group-functionalized adamantane (Ad_7NH2_2). (**b**) Zoomed-in view of the hydrogen bonds formed between the amino groups and water molecules. (**c**) Hole NTO density distribution of the excited states; the isovalue was $1\times 10^{-3}\;a_{0}^{-3}$. (**d**) Electron NTO density distribution; the isovalue was $2\times 10^{-4}\;a_{0}^{-3}$. The isovalues were selected based on the maximum density distribution. The excited-state energy was 5.258eV. The charge transfer to the water molecules ($q_{w}$) was 0.943.

To illustrate the characteristics of the charge transfer state, Figure 6a shows one of the selected MD snapshots. The zoomed view in Figure 6b highlights a water molecule that formed two hydrogen bonds with the two nearest amino groups. Therefore, the amino group contributed to complex solvation structures with water molecules. For the hole NTO densities, Figure 6c reveals that the density was localized on the nitrogen atoms. In contrast, the electron NTO density in Figure 6d was confined within the water shell and close to the surface amino functional group. Other snapshots revealed similar hole and electron distributions. Hence, the MD-based simulation of the solvation shell confirmed the conclusion derived from the simple water dimer model. The charge transfer occurred primarily from the nitrogen atoms to the nearest water molecules.

## 3. Theory and Methods

### 3.1. Electronic Structure Calculations

The electronic structure and properties of amino-decorated adamantane and water molecules were studied using density functional theory (DFT). The exchange correlation functional employed was the Coulomb-attenuated CAM-B3LYP [37], which is well suited for describing long-range and intermolecular charge-transfer processes. The basis set used was aug-cc-pVDZ [38,39], which includes diffuse functions to capture the diffuse surface states of adamantane and long-range charge transfer. In addition, Wang et al. [24] employed the CAM-B3LYP functional with the aug-cc-pVDZ basis set in a similar study. Their benchmarks show that this combination provides a more balanced description of the excited state compared with both range-separated and non-range-separated functionals. In particular, adding dispersion [40] as a perturbative correction to the total energy has a negligible impact on the computed excited states. Therefore, we used CAM-B3LYP without dispersion corrections. Furthermore, the aug-cc-pVDZ basis set shows convergence in excited-state energies, with the aug-cc-pVTZ basis set showing differences of about 1% for the same excited state.

Excited-state calculations were carried out via linear response time-dependent DFT (LR-TDDFT) using the same CAM-B3LYP/aug-cc-pVDZ set-up. For structures involving a shell of water molecules around amino-functionalized adamantane, the conductor-like polarizable continuum model (CPCM) [41] was used to account for the effect of water outside the solvation shell. All calculations were carried out using the ORCA software package (version 5.0.0) [42], while post-processing of the wavefunctions and population analysis were performed using the Multiwfn package (version 3.8) [43].

To investigate the charge transfer properties in different excited states, it is necessary to quantify the spatial separation between electrons and holes. In an excited state, an electron is promoted to a higher energy level, while the hole represents the positive charge left behind in the orbital originally occupied by the electron. The relative position of the hole and electron can therefore be used to classify different types of excitons. For example, in a charge transfer exciton, the hole is primarily localized on the adamantane molecule, whereas the electron is mainly localized on the water molecules. However, when both the hole and the electron are located either on adamantane or on the water clusters, no intermolecular charge transfer occurs.

Following one of our previous works [24], the natural transition orbitals (NTOs) [44] are used to characterize the spatial distributions of the electron and hole. The Hirshfeld population analysis [45] is then used to quantify the integrated electron and hole NTO densities on the atoms of the respective molecules.

For a charge transfer exciton, the amount of negative charge on the water cluster $q_{w}$ resulting from the separation of the hole and electron is defined as follows:

(1) $$\begin{array}{c} q_{w}=q_{w}^{e}-q_{w}^{h}, \end{array}$$

where $q_{w}^{e}$ represents the integrated electron NTO density for the water cluster (w) and $q_{w}^{h}$ is the corresponding integrated hole NTO density for the same water cluster.

Similarly, the amount of positive charge on the adamantane $q_{\mathrm{ad}}$ is defined by

(2) $$\begin{array}{c} q_{\mathrm{ad}}=q_{\mathrm{ad}}^{e}-q_{\mathrm{ad}}^{h}, \end{array}$$

where $q_{\mathrm{ad}}^{e}$ denotes the integrated electron NTO density for adamantane (ad) and $q_{\mathrm{ad}}^{h}$ denotes the corresponding integrated hole NTO density.

### 3.2. Molecular Dynamic Simulations

To characterize the solvation structure of water around amino-functionalized adamantane, we performed classical molecular dynamics (MD) simulations with the GROMACS (v2025.2) [46] package. The parameters for the solute were taken from the general Amber force field (GAFF), and atomic partial charges were generated with the AM1-BCC scheme in Antechamber (v24.8) [47]. The water was modeled with TIP3P [48].

A cubic simulation box with periodic boundary conditions was constructed such that the solute was at least 1 nm from each box face. The system was equilibrated in the NPT ensemble at P=1 bar and T=300 K until the box volume converged (17.0 nm^3^). The temperature and pressure were controlled using a Langevin thermostat and a Parrinello–Rahman barostat [49]. Production trajectories were then run in the NVT ensemble for 1 ns with a 4 fs timestep. Snapshots were extracted every 100 fs. For each snapshot, water molecules within a 7 Å sphere centered on the solute’s center of mass were extracted to define the hydration shell.

## 4. Conclusions

This study systematically explored the strategy of using amino group termination to reduce the energy gap of adamantane derivatives and particularly its impact on charge transfer to water molecules. The results show that amino functional groups can lower the LUMO energy levels, raise the HOMO energy levels, and reduce the energy gap, enabling better alignment with the solar spectrum and enhancing light absorption efficiency. Additionally, nitrogen atoms play a dominant role in charge transfer excited states, with the hole localized on nitrogen atoms rather than on carbon atoms. Therefore, nitrogen atoms act as the primary donors of solvated electrons rather than carbon atoms. However, excessive amino group functionalization can damage adamantane’s original NEA properties and limit the surface’s ability to emit electrons. Hence, maintaining a moderate level of amino functionalization is crucial to preserving NEA while optimizing the energy gap. Furthermore, water clusters near amino functional groups are prioritized to accept the photo-excited electron, with the closest nitrogen atoms acting as donors. Similarity, in a complex solvation environment, the charge transfer prioritizes the water molecules that are close to the nitrogen atoms. Thus, amino group functionalization offers a new approach to facilitating photo-excited charge transfer.

## Figures and Tables

**Table 1:** HOMO and LUMO energies, along with atomic charges, summarized for adamantane molecules with various surface functionalizations. The exact values of the HOMO and LUMO energies are dependent on the exchange correlation functional, which was CAM-B3LYP in our study. The molecular structures are illustrated in Figure 1. The Hirshfeld population analysis was performed on all atoms. The atomic charge values are averages for each atom type. The charges are in atomic units.

Molecule	HOMO (eV)	LUMO (eV)	C (*e*)	H (*e*)	N (*e*)
Ad	−9.131	0.249	−0.122	0.082	0
Ad_4NH2	−8.162	0.087	−0.026	0.088	−0.380
Ad_7NH2	−7.982	0.067	0.038	0.094	−0.355
Ad_7NH2_2	−7.410	0.070	0.022	0.091	−0.355
Ad_9NH2	−7.473	−0.004	0.060	0.094	−0.344
Ad_9NH2_2	−7.624	0.078	0.077	0.094	−0.339
Ad_10NH2	−7.355	−0.095	0.080	0.095	−0.337
Ad_11NH2	−7.265	−0.103	0.098	0.096	−0.331
Ad_11NH2_2	−7.524	−0.032	0.113	0.096	−0.329
Ad_13NH2	−7.786	−0.019	0.149	0.097	−0.322

**Table 2:** Properties of the lowest charge transfer states corresponding to the structures in Figure 3. For each structure, $q_{w}$ represents the magnitude of the electron transferred to the water molecules, while $q_{ad}^{N}$ denotes the electron-minus-hole density for all nitrogen atoms. Excitation energies of the lowest charge transfer states ($E_{ct}$) are given in eV, and transition dipole moments ($d_{ct}$) are in debyes (D). The order of the corresponding excited states is shown in the final column.

Molecule Structures	$q_{w}$	$q_{ad}^{N}$	$E_{\mathrm{ct}}$ (eV)	$d_{\mathrm{ct}}$ (D)	State
Ad_10NH2_CsC_a	0.426	−0.509	5.024	0.314	1
Ad_10NH2_CsC_af	0.420	−0.508	5.043	0.345	1
Ad_10NH2_CsC_f	0.368	−0.511	5.144	0.416	1
Ad_10NH2_CsC_l	0.312	−0.483	5.443	0.363	2
Ad_10NH2_CsC_r	0.333	−0.504	5.057	0.323	1
Ad_10NH2_CsC_rl	0.168	−0.484	5.164	0.330	1
Ad_7NH2_2_CsC_l	0.418	−0.494	5.056	0.508	1
Ad_7NH2_2_CsC_r	0.546	−0.549	5.705	0.193	3
Ad_7NH2_2_CsC_rl	0.418	−0.494	5.071	0.516	1

**Table 3:** Transferred charge ($q_{w}$), lowest charge transfer excitation energies ($E_{ct}$), and the order of the charge transfer states, with the distance between the water and nitrogen-doped adamantane increasing from $d_{0}$ Å to $d_{0}+4.0$ Å. The structure corresponds to the Ad_7NH2_2_CsC_lr configuration shown in Figure 3. The left half of the table presents the properties of the charge transfer (CT) excited states for the left water dimer, while the right half shows the corresponding properties for the right water dimer.

	CT Left			CT Right		
**$d_{0}$+**	$q_{w}$	**$E_{\mathrm{ct}}$ (eV)**	**State**	$q_{w}$	**$E_{\mathrm{ct}}$ (eV)**	**State**
0.0 Å	0.420	5.085	1	0.413	5.745	5
1.0 Å	0.360	5.154	1	0.160	5.718	4
2.0 Å	0.365	5.259	1	0.254	5.818	5
3.0 Å	0.252	5.331	1	0.118	5.891	5
4.0 Å	0.173	6.121	7	0.156	6.170	8

## Data Availability

Data are contained within the article. Further inquiries can be directed to the corresponding author(s).

## Appendix A

**Table A1:** Molecular Cartesian coordinates for Ad_7NH2_2_CsC_l, with Å as the coordinate units.

Atom	X	Y	Z
C	−0.26012	0.00000	1.50157
C	1.28888	−0.01809	1.46912
C	1.81839	1.23647	0.75085
C	1.79424	−1.27426	0.73188
C	−0.80559	0.00000	0.00000
C	1.29996	1.26362	−0.69938
C	1.28301	−1.26520	−0.72145
C	−0.27047	−1.31433	−0.74716
C	−0.25417	1.33188	−0.76265
C	1.79612	0.00490	−1.43526
N	−0.72494	1.21580	2.19689
N	−0.72275	−1.21019	2.23315
H	1.65393	−0.03150	2.50872
H	2.91690	1.20887	0.74509
H	1.52961	2.14134	1.29244
H	1.47769	−2.18425	1.25083
H	2.89227	−1.27328	0.72853
N	−2.27358	0.04046	0.04692
H	1.66101	2.15977	−1.21379
H	1.65613	−2.16345	−1.24021
N	−0.72165	−1.34732	−2.15932
N	−0.87230	−2.51389	−0.13860
N	−0.69825	2.60405	−0.18562
N	−0.67312	1.40890	−2.16211
H	2.89480	−0.00638	−1.44780
H	1.46132	0.03630	−2.47627
H	−2.61452	−0.92277	0.05531
H	−2.60265	0.44797	−0.82970
H	−1.38307	2.11902	−2.29801
H	−0.92560	0.50192	−2.54862
H	−0.46708	2.61517	0.80656
H	−1.71953	2.62010	−0.20607
H	−1.60758	−1.85003	−2.17759
H	−0.06316	−1.89269	−2.71387
H	−0.66134	−2.55454	0.86029
H	−0.49104	−3.33998	−0.60039
H	−0.51891	1.12070	3.19163
H	−1.74092	1.24678	2.09664
H	−1.73552	−1.14811	2.34296
H	−0.33125	−1.18213	3.17557
O	−5.42045	−0.07161	−1.54517
H	−5.42045	−0.06665	−0.59887
H	−5.42045	0.84285	−1.78865
O	−5.42045	−0.07161	1.37114
H	−4.67166	0.18477	1.88988
H	−6.16925	0.18477	1.88988
